# Adapting Protocols or Models for Use in Insulin-Requiring Diabetes and Islet Transplant Recipients

**DOI:** 10.3389/fendo.2021.611512

**Published:** 2021-07-16

**Authors:** Glenn M. Ward, Jacqueline M. Walters, Judith L. Gooley, Raymond C. Boston

**Affiliations:** ^1^ Department of Endocrinology and Diabetes, St. Vincent’s Hospital, Fitzroy, VIC, Australia; ^2^ Department of Clinical Biochemistry, St. Vincent’s Hospital, Fitzroy, VIC, Australia; ^3^ University of Melbourne Department of Medicine, St. Vincent’s Hospital, Fitzroy, VIC, Australia; ^4^ School of Veterinary Medicine, University of Pennsylvania, Philadelphia, PA, United States

**Keywords:** mathematical modeling, type 1 diabetes mellitus, islet transplantation, insulin secretion, insulin sensitivity, minimal model, C-peptide model

## Abstract

The authors’ perspective is described regarding modifications made in their clinic to glucose challenge protocols and mathematical models in order to estimate insulin secretion, insulin sensitivity and glucose effectiveness in patients living with Insulin-Requiring Diabetes and patients who received Pancreatic Islet Transplants to treat Type I diabetes (T1D) with Impaired Awareness of Hypoglycemia. The evolutions are described of protocols and models for use in T1D, and Insulin-Requiring Type 2 Diabetes (T2D) that were the basis for studies in the Islet Recipients. In each group, the need for modifications, and how the protocols and models were adapted is discussed. How the ongoing application of the adaptations is clarifying the Islet pathophysiology in the Islet Transplant Recipients is outlined.

## Introduction

In this article we describe the evolution of the modifications we made in our clinic to glucose challenge protocols or mathematical models of insulin secretion, insulin sensitivity and glucose effectiveness, in order to study these parameters in patients with Insulin-Requiring Type2 Diabetes (T2D) and Type 1 diabetes (T1D), including T1D patients who have received Islet Transplants to treat their severe recurrent hypoglycemia and impaired awareness of hypoglycemia. This includes fitting of the Minimal Model of Bergman et al. (1) to Intravenous Glucose Tolerance Tests (IVGTT), and of the ISEC model to Oral Glucose Tolerance Tests (OGTT). We revisit the adaptations that were made for use in T1D, and Insulin-Requiring Type 2 Diabetes (T2D) as it helps to build a cohesive account of the work in our clinic aimed at studying the pathophysiology of insulin secretion and insulin action in the Islet Transplant Recipients. In each group we consider what issues were encountered, how we overcame them, and why we chose to adapt the protocols or models.

## Estimation of Insulin Sensitivity From Intravenous Glucose Tolerance Tests in T1D

### Modification of Minimal Model to Apply to Stepped Insulin-Modified IVGTT in T1D

The Minimal Model of Bergman et al. (1) consists of a Minimal Model of Glucose Disappearance (the “Glucose Minimal Model”, gMM) [2, Equations 1 & 2] and a Minimal Model of Insulin Kinetics (the “Insulin Minimal Model”, iMM) [3, Equation 3].

The gMM can be fitted to plasma glucose and insulin data from an IVGTT to simultaneously estimate Insulin Sensitivity (Si, increase in fractional glucose disappearance per unit increase in plasma insulin) and Glucose Effectiveness (Sg, ability of glucose per se to enhance its own disappearance independent of an increment in plasma insulin above basal).The iMM can be fitted to a IVGTT to estimate beta-cell responsiveness to glucose: first-phase responsivity (Phi1, amount of insulin (per unit volume) that can be accounted for by an assumed initial injection, per unit change in plasma glucose); and, second-phase responsivity (Phi2, the proportionality factor between glucose and the rate of rise of insulin secretion). Software to fit both models to IVGTT data includes Minmod (4). An alternative method used in our laboratory utilizes the SAAM modeling program to fit the model equations 1 to 3 as described (5).

1 dG/dt=−(p1+X(t))*G(t)+p1*Gb

2 dX/dt=−p2.X(t)*p3(I(t)−Ib)

3 dI/dt=−nI(t)+γ(G(t)−h).t

Where: G(t) and I(t) are the time courses of glucose and insulin in plasma following a rapid intravenous injection of glucose; Gb and Ib are basal levels; X(t) is the insulin effect on net glucose disappearance; p1 is glucose-mediated glucose disposal; p2 is insulin degradation; p3 is insulin action; n is the insulin clearance; γ is the proportionality factor between glucose concentration and the rate of increase of second phase insulin secretion for plasma glucose levels G(t) that exceed h, the threshold glucose level.

Although this method successfully accommodated data from healthy subjects and a variety of pathological states in fitting the gMM, we found it could not fit data from many patients with Type 1 diabetes (T1D) because: there was insufficient insulin input into the model as commented by Pacini and Bergman (4); and, insulin antibodies in many T1D interfered with the measurement of plasma insulin. In addition, Godsland and Walton showed that the success rate of minimal model analysis is reduced if the glucose does not return to baseline (6). We subsequently applied a modification of the gMM, which included a model of the exogenous insulin infusions, to IVGTT data from T1D subjects who had undergone a modified exogenous insulin protocol (7). This aimed to achieving sufficient insulin input to the model to regularize the glucose disappearance curves with sufficient features in the glucose curves to enable successful identification of the parameters (4). We also addressed the frequent presence in T1D of insulin antibodies which interfere with the insulin radioimmunoassay, by measuring plasma free-insulin after precipitation of bound insulin according to the method of Nakagawa et al. (8) using polyethylene glycol precipitation before freezing in order to avoid disturbing the equilibrium between free and antibody-bound insulin (9). In a recent study using a dextran-coated charcoal insulin assay (10) in our clinic, insulin antibodies were detected in a very low percentage in non-diabetic subjects, but in 50% of T1D, and 74% of Islet Transplant Recipients (manuscript in preparation).

**Figure 1:**
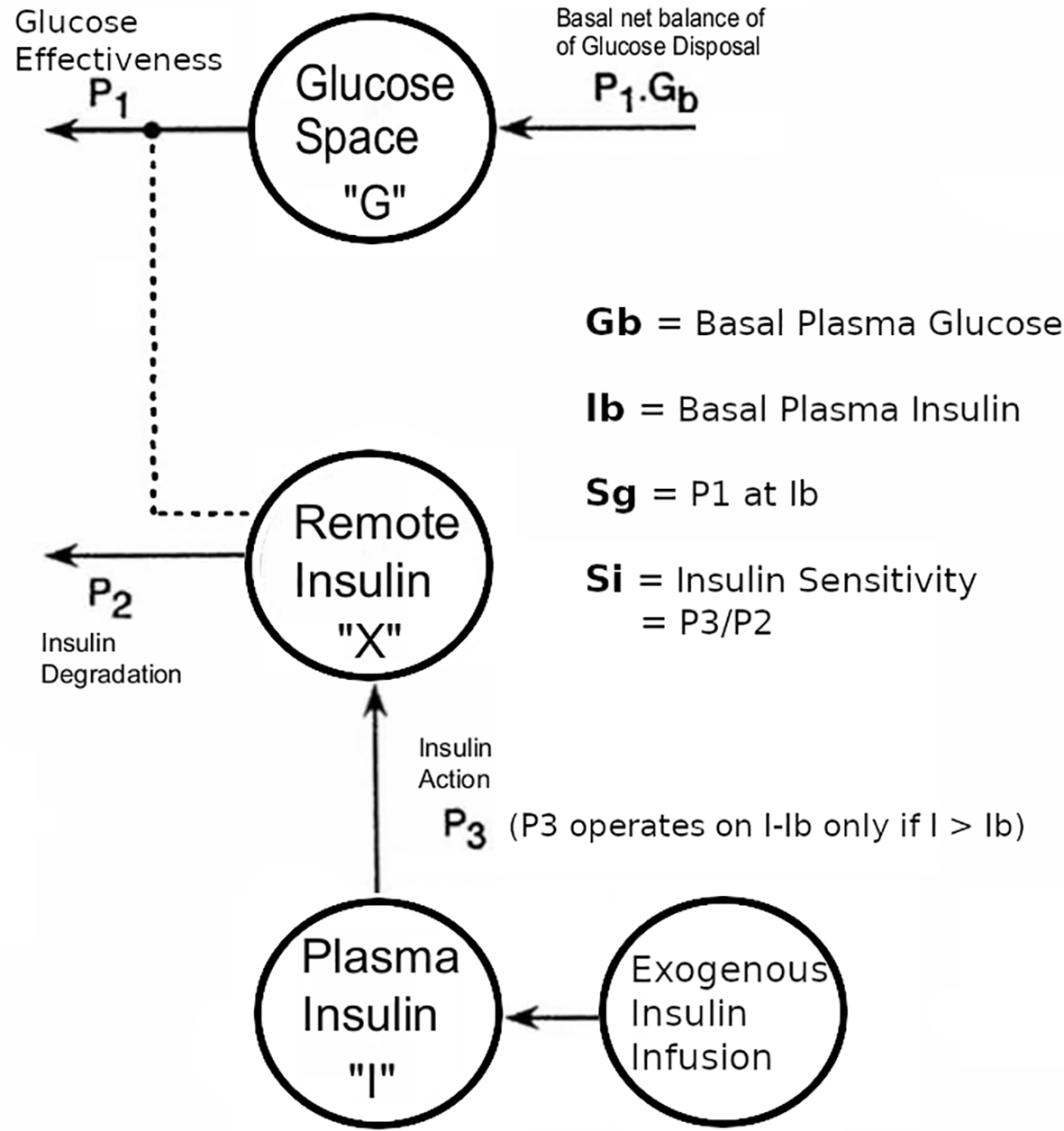
Modifications to the Bergman minimal model of the intravenous tolerance test, aimed at enabling the model to be applied to data from T1D subjects in whom endogenous insulin secretion was minimal compared with the exogenous insulin infusion. Note that the endogenous coupling of the plasma insulin response to plasma glucose that was used in our modeling of non-diabetic subjects (5) was replaced with an external insulin supply represented by an additional compartment, as described in reference 7. This figure has been reproduced in a modified form from 7 with permission.

**Figure 2:**
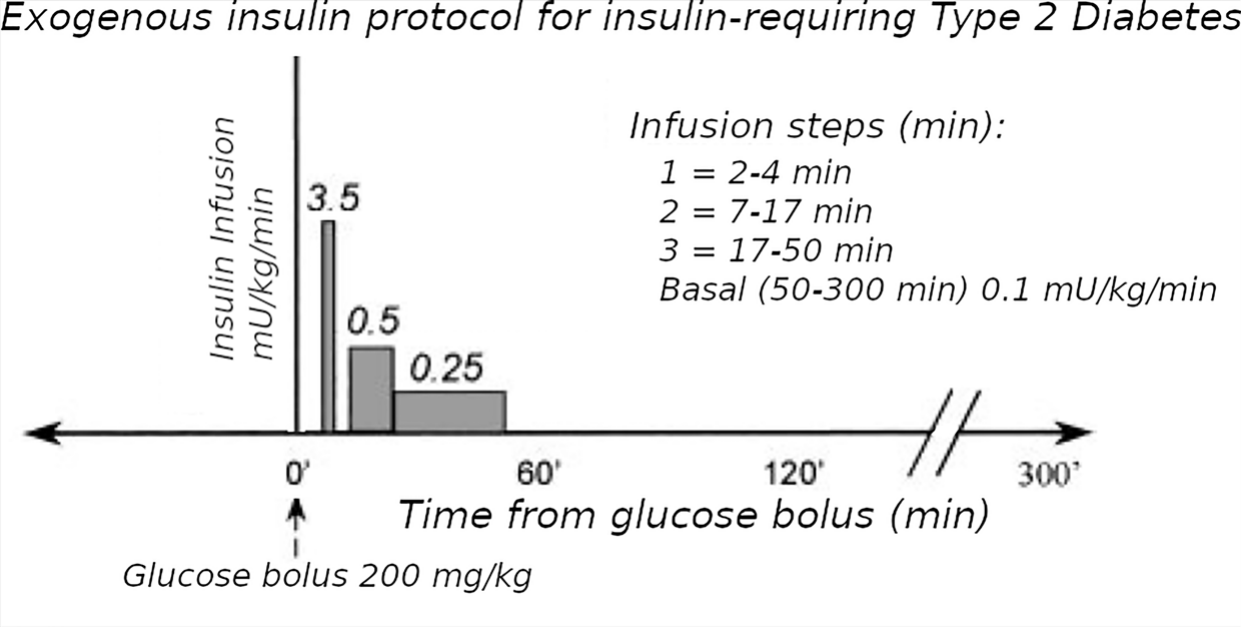
Exogenous insulin protocol in T2D. This figure has been reproduced in a modified form from 11 with permission. It shows the steps of insulin infusion used in the T2D protocol, but also is similar in principle to the stepped protocol used for T1D.

To simulate a more physiological insulin profile during the IVGTTs in T1D, stepped exogenous insulin was infused with the total dose modified to achieve near-normal glucose disappearance (K_g_). The stepped protocol aimed at approximating the insulin profile seen during an IVGTT in healthy normal weight subjects, derived by simulation of insulin disappearance kinetics. The final pattern of insulin infusion (analogous to **Figure 2**) was: 2-4 min = 14 mU of insulin per kg; 7-16min = 1 mU/min/kg; 17-50 min = 0.5 mU/min/kg; 51-180 min = rate estimated to maintain basal euglycemia based on the previous overnight insulin requirement. A model of the insulin infusion was added to the Minimal Model to estimate Si and Sg (**Figure 1**) (7), in 8 T1D subjects (age 21-38 y, BMI 20-26 kg/m^2^) *versus* 17 healthy control subjects(20-37 y, 19-25 kg/m^2)^)’.

The exogenous insulin protocol in T1D IVGTTs achieved near-normal plasma free-insulin levels: first-phase = 62 ± 9 SE mu/L; second-phase = 34 ± 9 mu/L, and K_g_ was normal at 1.3 ± 0.29 min^-1^ x 10^2^ (7). Using the modified model and protocol as described (7) (**Figure 1**), we found T1D v controls: Si = 2.5 ± 0.6 v 8.3 ± 1.5 min^-1^.mU^-1^.L^-1^ x 10^4^; Sg = 1.6 ± 0.5 v 2.6 ± 0.2 min^-1^ x 10^2^; P <.05 Mann-Whitney, Fractional Standard Deviation < 0.5. Given that protocols used in the diabetic subjects were different from the normal subjects, it must be carefully considered whether the estimates are robust to structural perturbations. Nevertheless we examined the robustness by the following experiments. Using IVGTT during basal insulin infusion as described by Ader et al. (12), Sg was verified to be similar by this technique in the same T1D subjects (1.0 min^-1^ x 10^2^, p = NS, Mann-Whitney) (7). We verified Si by comparison to a previous euglycemic clamp study where Si was 4.2 ± 1.0 min^-1^.mU^-1^.L^-1^ x 10^4^ in a similar T1D group (p =NS, Mann-Whitney) (13). Therefore we concluded that the estimates were sufficiently robust to structural protocol perturbations, particularly if experiments could be designed using subjects as their own controls with the same protocol, as will be exemplified in the next three sections.

### Alternatives to Stepped Insulin-Modified IVGTT in T1D

With regard to the stepped insulin infusions in the protocol, there can be alternative approaches that may not require as significant overhauls to the protocol. With the increasing use of continuous subcutaneous insulin pumps, a basal intravenous insulin infusion from 51 to 180 minutes may not be necessary during the IVGTT if the basal pump rate is continued, providing insulin assays are used with specificity for analogue insulin (14, 15), such as research-grade assays with defined cross-reactivity with analogue insulin. The alternative approach of cessation of insulin pumps and switching to a long acting insulin prior to IVGTT may see unpredictable declines in plasma insulin (16) and may risk hypoglycemia while fasting. Another alternative regimen has been switching to a night-time dose of intermediate insulin, but this is sometimes associated with the need for an intravenous basal insulin infusion during the IVGTT.

### Stepped Insulin-Modified IVGTT Protocol With Epinephrine in T1D

A practical example and test of the robustness of the use of the Stepped IVGTT protocol (A.1) was our study to explore the relative roles of Sg and Si in the observed impairment of glucose disposal with epinephrine infusion in T1D (17). An eight-fold rise in plasma epinephrine was achieved by intravenous delivery at 25ng/kg/min for 5.5 hours (EPI), in 7 non-obese young adult T1D patients, none of whom were on insulin pumps, but who had a basal overnight insulin infusion (12mU/kg/hr) with euglycemia maintained by adjustment of intravenous glucose. At 2.5 hours the IVGTT was performed with the stepped exogenous insulin protocol and analyzed as described (A.1) (7). Each subject had in random order a control (CTR) infusion of basal insulin prior to the IVGTT. Elevation of plasma epinephrine caused: impaired glucose disposal (Kg) (EPI 0.59 ± 0.1 *vs* CTL 1.91 ± 0.33 min ^-1^ x 10^2^, p<0.02 Mann-Whitney), associated with a marked impairment of Si (EPI 0.9 ± 0.5 *vs* CTR 7.03 ± 3.2 min^-1^.mU^-1^.L x 10^4^, p<0.05 Mann-Whitney); but, no impairment of Sg (EPI 2.5 ± 0.2 *vs* CTR 3.1 ± 0.5 min^-1^ x 10^2^) [p=NS Mann-Whitney]. These experiments indicated that physiological epinephrine elevation in T1D impairs Si but not Sg (17). Therefore, even in patients not on insulin pumps, the baseline insulin infusion during the IVGTT is able to effectively maintain basal glucose levels despite perturbation by Epinephrine.

### Stepped Insulin-Modified IVGTT Protocol With Pulsatile Insulin Infusions in T1D

Another practical example of the robustness of protocol A.1 in T1D subjects, is our study of pulsatile insulin infusions in which, therapeutic levels of intravenous pulsatile insulin were compared with continuous intravenous insulin, at matching levels in T1D subjects (18). Of the 11 young non-obese T1D subjects, 4 had detectable fasting plasma C-peptide (40 ± 20SE pmol/L) and 5 had diabetes-duration above 10 years. Insulin was delivered intravenously at 12 mU/kg/h overnight for 17h, either as 40-second pulses every 13 minutes (PI) or continuously (CI), and euglycemia was maintained during the overnight fast by adjustable intravenous glucose. The next morning a fasting IVGTT was performed and analyzed as above (A.1).

The hypoglycemic effect of PI *versus* CI, estimated by glucose infusion rates, was approximately doubled in the 6 subjects with duration less than 10 years, (PI *vs* CI, 7.5 ± 2.7 *vs* 3.2 ± 0.6 µmol/kg/min p <0.05 Mann-Whitney) but did not differ in the 5 subjects with duration over 10 years (PI *vs* CI, 5.8 ± 2.4 *vs* 4.7 ± 2.2 µmol/kg/min). Insulin sensitivity from analysis of the IVGTT data was uniformly increased after PI *versus* CI with duration under 10 years (PI *vs* CI, 4.9 ± 1.4 *vs* 3.0 ± 1.0 min^-1^.mU^-1^. L x104). After 10 years diabetes duration insulin sensitivity was uniformly greater with CI than with PI (PI *vs* CI, 0.3 ± 0.1 *vs* 2.9 ± 1.6 min^-1^.mU^-1^. L x104, p <0.05 Mann-Whitney).

We concluded that, prolonged pulsatile *versus* continuous intravenous insulin resulted in a significant increase in hypoglycemic effects and insulin sensitivity in T1D with diabetes duration up to 10 years the differential effect of PI was dependent on duration of diabetes. This indicates that the use of a basal insulin infusion during the IVGTT in T1D patients who are not on insulin pumps, is effective in maintaining the constant pattern of glucose levels at the end of the IVGTT, despite pulsatile insulin infusions being used.

## Estimation of Insulin Secretion and Insulin Sensitivity in T1D After Islet Transplantation

### Pancreatic Islet Transplantation in T1D

Pancreatic islet transplantation (IT) is an established clinical treatment for people with T1D, who suffer with severe hypoglycemia unawareness. Islets are obtained from the pancreas of a deceased organ donor, purified and then transfused into the portal vein of the recipient. Restoring natural islet function improves glycemic control and can markedly reduce hypoglycemia. Transplant recipients need life-long immunosuppression to prevent rejection-mediated cell loss.

In order to apply both iMM and gMM to analyze IVGTTs in Islet Transplant Recipients (ITR), we found there was a need to further modify the protocol and model compared to the T1D analyses, building on the investigations done on T2D IVGTTs discussed below.

### Extension of Modifications of Protocol and Model to T2D

**Figure 3:**
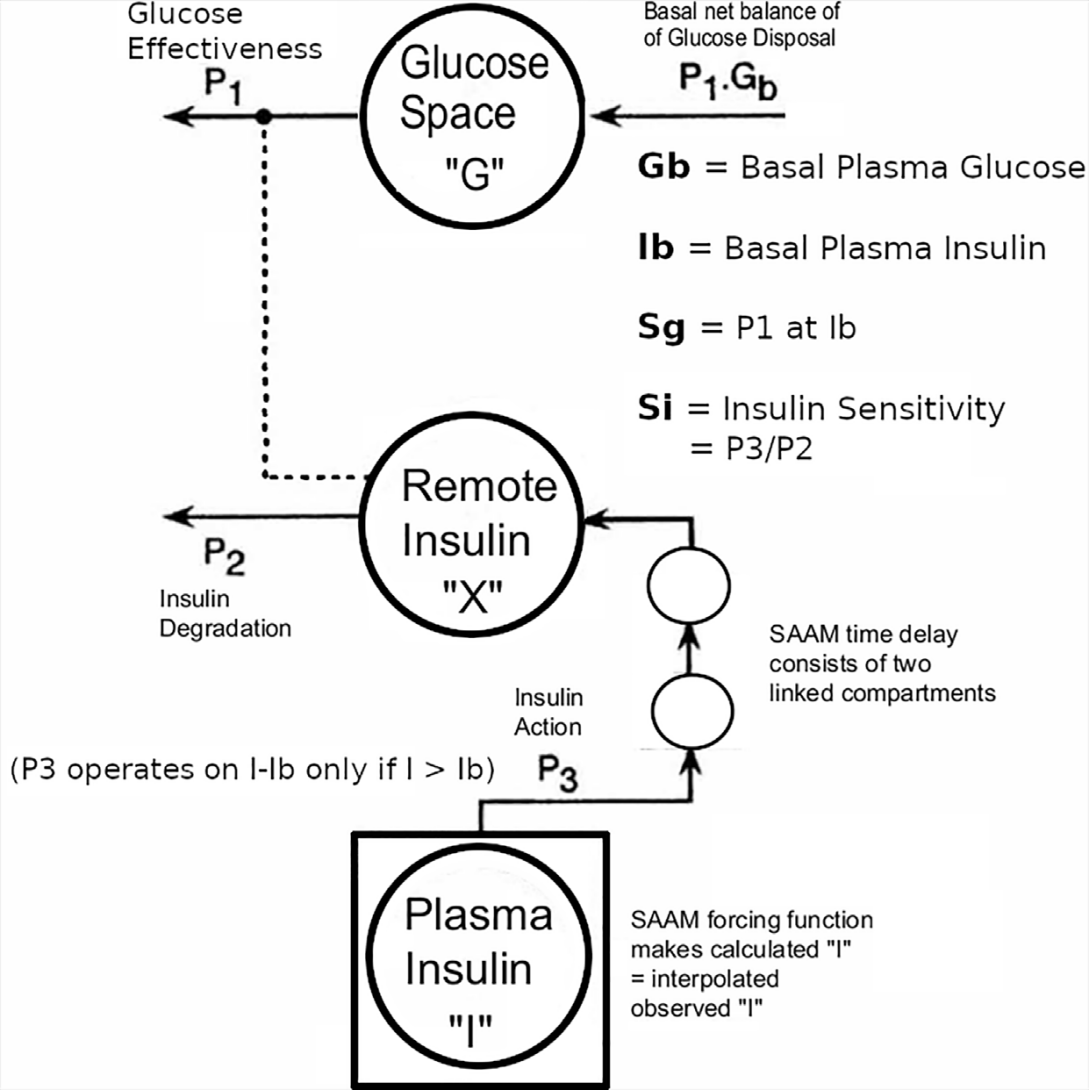
Modification of Minimal Model to accommodate T2D data with the physiological exogenous insulin protocol. This figure has been reproduced in a modified form from 11 with permission. The IVGTT glucose and insulin data were analyzed using the glucose model of Bergman et al. as described (5) using the simulation, analysis, and modeling program SAAM (6). Except there were the following modifications: The square around compartment “I” indicates that the observed plasma insulin concentrations drive the system, and an additional element that is the equivalent of two linked compartments represents a time delay in the transfer of insulin into the remote insulin compartment (X). The program SAAM generates and numerically solves the differential equations without the need to supply explicit differential equations representing the time delay element.

The Minimal Model of Bergman et al. could only be used in early studies of T2D subjects if they had sufficient endogenous insulin secretion. An adequate increase during the IVGTT in the AUC of insulin levels was necessary to be able to fit the Minimal Model. To overcome this limitation, exogenous insulin protocols have been used in IVGTTs in T2D to enable the Minimal Model to estimate Si and Sg (19, 20). Also, in insulin-requiring T2D, the baseline insulin levels may need to be maintained during the IVGTT when insulin secretion is low. Therefore we adapted protocol A.1 with basal insulin infusion during the IVGTT for use in insulin-requiring T2D. However, we found (11) that the estimates were affected by the doses of insulin used: either Welsh et al. (19) or Taniguchi et al. (20). Because of this, we developed a “minimal disturbance” approach to estimating Si and Sg in T2D. To avoid supra-physiological peak glucoses in T2D with elevated fasting glucose, we used a reduced glucose load (200 mg/kg). In order to compensate for endogenous insulin secretion in T2D the T1D Stepped insulin infusion rates (A.1) were reduced by 50% (**Figure 2**). In a series of 8 T2D patients, 5 of whom were insulin-requiring, data from this approach were analyzed either using the unmodified Minimal Model of Bergman (BMM), or a modified model (MMD) with an additional element (in this case DT18 in SAAM terminology) representing a time delay in the transfer of insulin into the remote insulin compartment (X). As described in (11) (**Figure 3**), the program SAAM compiles the model “deck” and generates and numerically solves the differential equations without needing explicit differential equations representing DT18.

Adaptation by adding the delay element improved identification of Si and Sg from 37.5% (BMM) to 100% (MMD) in this largely insulin-requiring T2D group. Si in these T2D subjects was lower than normal(1.86 ± 0.60 v 8.65 ± 2.27 min^-1^.mU^-1^.L x 10^4^,p <.01 Mann-Whitney). The reduced Si values were confirmed in this T2D group with 2-stage euglycemic clamps (Si CLAMP = 2.02 ± 0.42 min^-1.^mU^-1^.L x 10^4^, p > 0.4 *vs* IVGTT Mann-Whitney). Sg was not significantly reduced (2.00 ± 0.25 T2D v 1.55 ± 0.26 normal, min^-1^ x 10^2^). Use of the delay in normal subjects did not improve the fit.

These results suggest that insulin action at physiological insulin levels in insulin-requiring T2D may not be a single phase, possibly due to impaired trans-capillary endothelial transfer.

In the process of protocol selection, we found that, since our protocol could accommodate insulin requiring T2D, some needed free insulin assay, and some may need basal insulin infusions during the IVGTT. These studies indicate that, in T2D with minimal insulin secretion such as insulin-requiring T2D, we would recommend using an IVGTT protocol with basal insulin infusion during the IVGTT.

### Delay in Insulin Secretion During IVGTT and OGTT After Islet Transplantation in T1D

Selection of the exogenous insulin protocol for IVGTT for Islet Transplant Recipients (ITR) in our clinic was informed by our previous adaptations in T1D and T2D. Free insulin assays were used if insulin antibodies were detected in individual subjects, and this was necessary in about 75% of cases. Although ITRs have features similar to T2D, their insulin sensitivity was more similar to T1D so that a more standard exogenous insulin protocol could be used, and without the need for a delay element in the modeling. Approximately 50% of ITRs became insulin independent and had better homeostasis of the basal glucose and insulin. In these subjects we were able to model IVGTTs without exogenous insulin supplementation as described (5). Alternatively, many insulin dependent ITRs used insulin pump therapy, enabling stabilization of the baseline state by continuing the insulin pump together with the Taniguchi exogenous insulin protocol (20).

**Figure 4:**
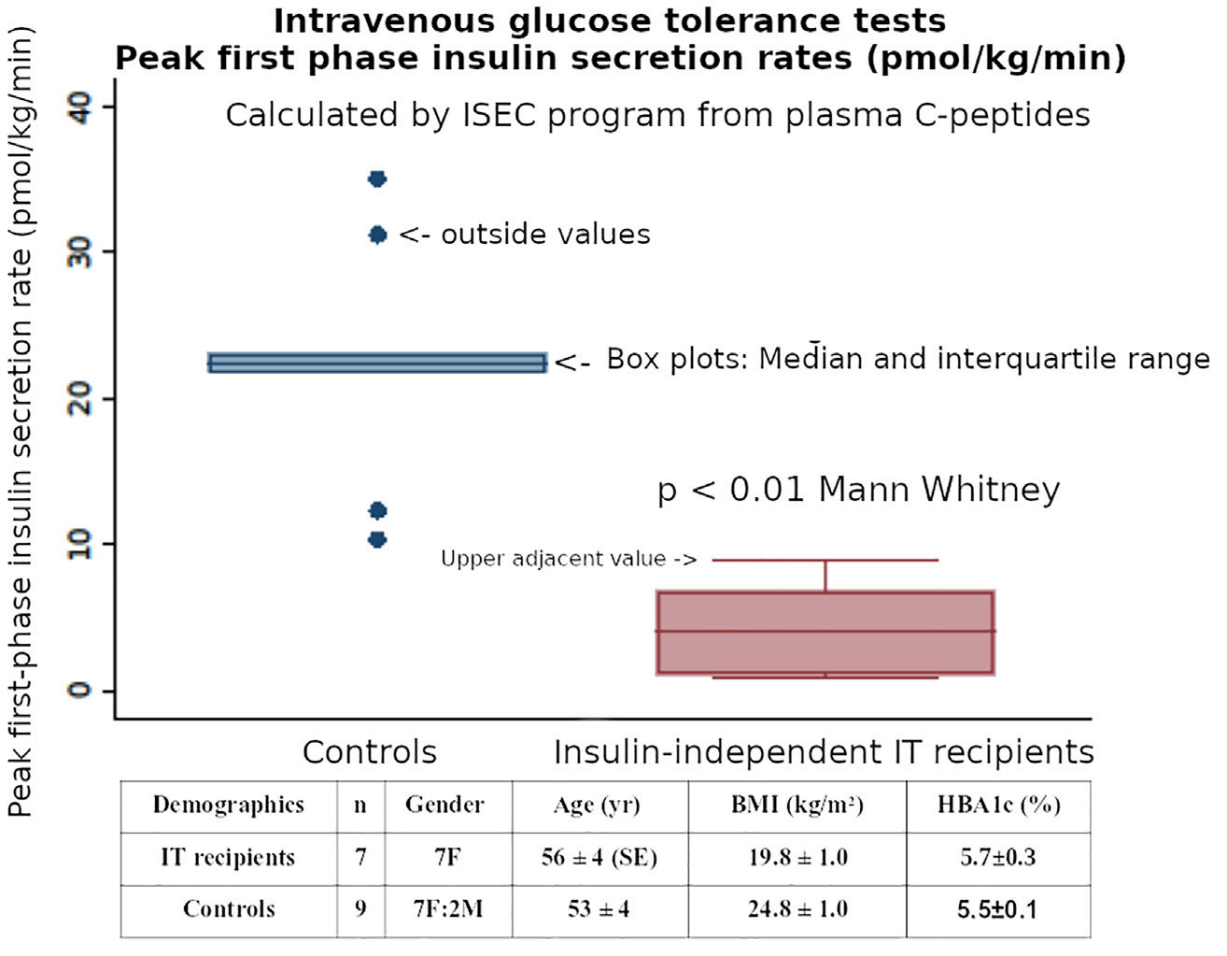
The post-transplant first-phase insulin secretion (Phi1) during an IVGTT, when calculated by ISEC analysis of plasma C-peptide levels, was reduced in insulin independent Islet Transplant recipients - compared with matched healthy controls.

In the ITRs we chose also to model plasma C-peptide responses during the glucose challenges to estimate Insulin Secretion Rates by the ISEC methodology (21). We aimed to maximize the information to further investigate the pathophysiology of insulin secretion by the transplanted islets. We reported deficient first-phase insulin secretion (Phi1) during IV glucose tolerance tests and greater restoration of second compared with first phase insulin secretion after successful islet transplantation, with maintained Si despite being on immunosuppression regimens after islet transplantation (22), indicating that comprehensive estimates of insulin secretion capacity (first and second phases, and DI) with the Non-insulin modified IVGTT (NIM-IVGTT) have an significant role in metabolic monitoring after islet transplantation in subjects who are insulin independent. This finding contrasted with other studies using IVGTT which showed that islet transplantation can restore first-phase insulin secretion to the normal range (23, 24). Further studies of the insulin independent ITR patients included C-peptide ISEC analyses and our preliminary data confirms a reduction in first phase insulin secretion rates (**Figure 4**), and this would also indicate that our previous results with plasma insulin Phi1 (22) were not reduced secondary to binding of secreted insulin by circulating insulin antibodies.

We extended our studies of the delays of early secretion of insulin to include Oral Glucose Tolerance Tests (OGTT) because this test included the incretin effects (25, 26) which is a potentially important element in the ITR group, and we found that the incretin effect was reduced in our cohort of ITR (25, 26). We found however that one practical drawback, was the difficulty of using OGTTs in the insulin-dependent subset of ITRs because of the lack of standardization of exogenous insulin protocols during the OGTT, which would be needed to avoid undesirable hyperglycemia during the tests in the insulin-dependent group. It is also difficult to model the insulin responses in this group because of the low insulin responses limit the ability to fit the model to the data (6). We therefore focused our OGTT studies on the insulin-independent ITRs which make up about 50% of the ITRs in our clinic in accordance with other clinics who use the standard Edmonton Islet Transplant Protocol (27).

Our studies using OGTTs in Islet Transplant recipients (25) indicated that there is a delay in insulin secretion rates which may be related to factors such as incretin function (26). Our OGTT studies also demonstrated a normalized suppression of free fatty acids in islet transplant recipients despite this delay in insulin secretion (28).

Our ITR subjects were 7 T1D patients (Group A, gender 7F, age 56 ± 4 SE yr, BMI 19.8 ± 1.0 kg/m2, T1D duration 46 ± 10 yr.) who had achieved insulin-free status following IT as described in our Multicenter Trial (29). The detailed exclusion and inclusion criteria for the transplants were as described (29) but the key criteria were T1D patients with life-threatening severe recurrent hypoglycemia and impaired awareness of hypoglycemia, but who were suitable for immunosuppression. They were compared to 9 matched non-diabetic controls as described (25). (Group B, 7F:2M, 53 ± 4 yr., BMI 24.8 ± 1.0 kg/m2). All subjects had both OGTTs and IVGTTs. The clinical investigations described here were carried out with the approval of the Institutional Human Research Ethics committee at St Vincent’s Hospital Melbourne. Within 6 months of gaining insulin-independence, 75-gram 4-hour OGTTs and 200mg/kg IVGTTs were performed in the 7 insulin-independent T1D islet transplant recipients and compared to the similar non-diabetic healthy subjects who had both an OGTT and an IVGTT within 6 months of each other. The groups A & B had similar glucose levels (transplant recipients *vs* healthy non-diabetic subjects: mean HBA1c 5.7 ± 0.3SE *vs* 5.5 ± 0.1% p=NS; and similar insulin sensitivity HOMA2-S% 117 ± 28 *vs* 83 ± 8 p=NS Mann-Whitney).

Plasma glucose, insulin and C-peptide were measured at 30 minute intervals during the OGTTs, and as previously described (7) during the IVGTTs.

Insulin secretion rates were calculated by using ISEC to fit a model of C-peptide kinetics to the plasma C-peptide concentrations during both IVGTTs and OGTTs (21). Using these Insulin Secretion Rates (ISR) during the IVGTTs, the initial post-transplant first-phase insulin secretion (Phi1, peak value in first 10 minutes) was reduced in recipients compared with healthy non-diabetic subjects (median [Interquartile Range] 4.1 [1.1-6.78] *vs* 22.4 [21.8-23.1] pmol/kg/min, respectively, p<0.01 Mann Whitney). (**Figure 4**). Using the ISR during the first 30 minutes of the OGTT to calculate first-phase insulin secretion (oPhi1, as the increment in ISR per increment in glucose) also showed a reduction in the recipients *versus* healthy non-diabetic subjects (0.43 [0.26-1.11] *vs* 2.32 [1.41-2.59] pmol/kg/min per mmol/L, respectively, p<0.01 Mann Whitney). The above data on Phi1 and oPhi1 were not normally distributed so non-parametric tests were used.

**Figure 5:**
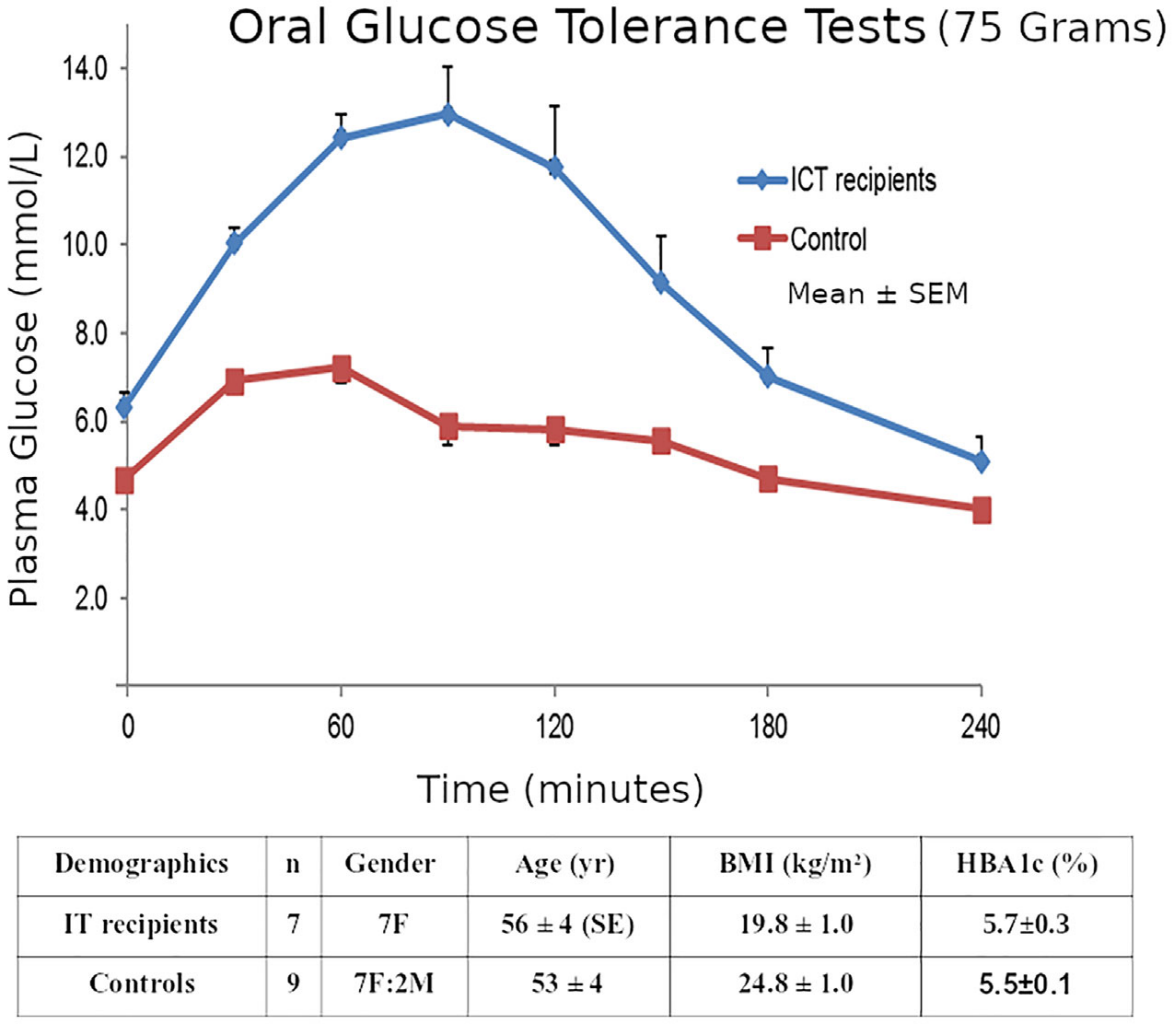
Mean ± SE plasma glucose concentrations during 75g OGTTs in the 7 Insulin-independent Islet Transplant Recipients, and in the 9 Nondiabetic Controls.

Although the transplant recipients’ mean OGTT 2-hour glucose was elevated (13.8 ± 1.7 mmol/L), 2 recipients were classified as non-diabetic (<11.1), and all recipients’ glucoses returned to baseline (5.8 ± 1.2) by 4-hours. (**Figure 5**).

**Figure 6:**
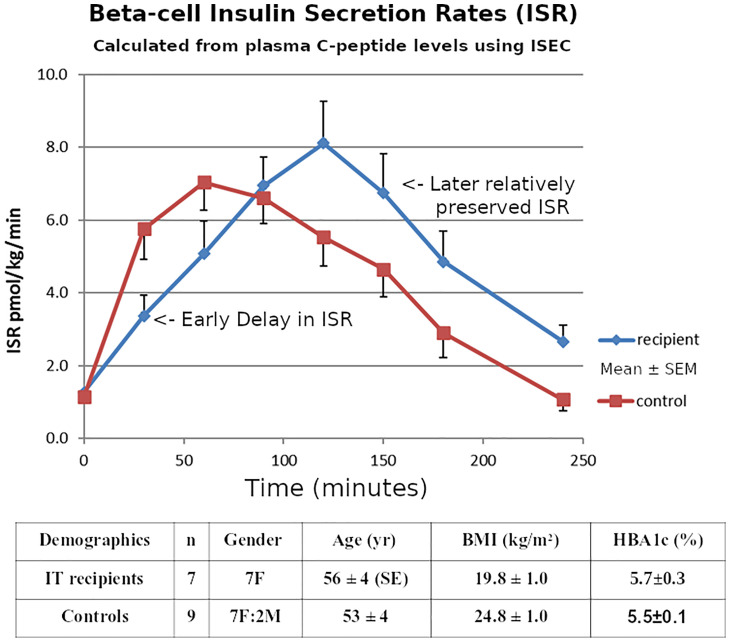
Mean ± SE Pre-hepatic insulin secretion rates estimated from the plasma C-peptide concentrations by deconvolution using the program ISEC, during 75g OGTTs in the 7 Insulin-independent Islet Transplant Recipients, and in the 9 Nondiabetic Controls.

ISEC analysis of the plasma C-peptide allowed estimation of Insulin Secretion Rates (**Figure 6**), showing a delay in early insulin secretion with clear improvement in the latter half of the OGTT. The relationship of this improvement to incretin effects requires further investigation.

Only measuring early insulin release during OGTTs could underestimate later secretion by ~30% in islet transplantation, correlating with our previous report using IVGTTs (22). The good control of HBA1c in the recipients despite the delayed early secretion could be related to the portal route of transplantation, or might reflect the dietary preferences of the recipients.

However, our findings support a role for also testing the early secretion using IVGTT. When C-peptide-derived Insulin Secretion Rates during the IVGTTs were estimated using ISEC, the initial post-transplant first-phase insulin secretion was reduced in recipients compared with healthy non-diabetic subjects. This confirms our previous conclusion based on plasma insulin concentrations (22), indicating that the reduced Phi1 was not caused by binding of secreted insulin by insulin antibodies.

Reduced IVGTT Phi1 after islet transplantation could reflect the same factors as the delayed insulin secretion during OGTT. For example it has been suggested that increased beta-cell overdrive could cause depletion of readily-releasable insulin stores. This marker of beta cell dysfunction provides a parameter that could be monitored in addition to indices of beta cell mass. Alternatively, these reduced insulin responses could represent recurrence of autoimmune beta cell damage similarly to that observed with reduced first phase insulin responses in pre-type 1 diabetes patients (30). Further studies are underway in our clinic with greater number of patients to clarify the significance of this delay in insulin secretion and its relationship of the pathophysiology of the decline in islet function after ITR.

### Future Directions in IVGTT and OGTT After Islet Transplantation in T1D

Future evaluation of beta cell function in islet transplant recipients would be improved by better understanding of the interaction of insulin sensitivity, glucose effectiveness and the parameters of beta cell secretion of insulin. It is well accepted that insulin secretion and sensitivity are best interpreted together, because of the hyperbolic relationship between these two parameters (31), and that an improved measure of beta cell function is obtained by calculating the “disposition index” (i.e. the “insulin sensitivity-adjusted beta cell function”) (31). The other less well-understood interaction is the degree to which exogenous insulin given during IVGTTs can directly suppress endogenous insulin secretion, independent of the impact upon glucose levels, due to the feedback loop of circulating insulin on its own secretion (32). The current methods to correct for this effect mainly involve omitting of data during exogenous insulin supplementation but would benefit from standardization. Other methods of data analysis such as Bayesian hierarchical analysis could be explored that could improve parameter estimation (33), although it would need to be confirmed whether this would avoid the need for optimized exogenous insulin protocols during the IVGTT. Alternatively algorithms developed for closed-loop subcutaneous insulin pumps (34) and successfully used in exercise perturbation studies (35), may allow adaptation to intravenous glucose monitoring and insulin delivery, which may allow real-time adjustment of insulin infusions during IVGTT or OGTTs. This would optimize the glucose decay curves and therefore the ability to identify parameters for Sg and Si (6).

## Overall Summary

We have presented our perspective of the application of the Mathematical Models to the analysis of intravenous and oral glucose challenges in Type I diabetes. The modifications of the protocols necessary to apply these models also to Type I diabetes patients who have received Islet Transplants were elaborated. Islet Transplant Recipients represent a pathophysiological state that is similar to T2D, but has some distinct differences. These differences are exposed by application of the modified protocols and models of insulin secretion and action. In our preliminary studies, IVGTT *versus* OGTT parameters provided additional insights into the pathophysiology of transplanted islets, reflecting beta cell dysfunction rather than only monitoring beta cell mass (23) in Islet Transplant Recipients. Further evaluation with greater numbers of ITR is required to explore the relevance of delayed early insulin secretion in determining survival of transplanted islets.

## Data Availability Statement

The raw data supporting the conclusions of this article will be made available by the authors, without undue reservation.

## Ethics Statement

The studies involving human participants were reviewed and approved by St Vincent’s Hospital Human Research Ethics Committee. The patients/participants provided their written informed consent to participate in this study.

## Author Contributions

GW, JG, JW, and RB collaborated on the investigations that form the basis of this perspective. GW wrote the initial draft based on previous discussions with the coauthors, and JG, JW, and RB made substantial contributions to revisions of it. All authors contributed to the article and approved the submitted version.

## Funding

The work discussed in this manuscript was supported by grants from Diabetes Australia Research Foundation, St Vincent’s Hospital Research Foundation, National Health and Medical Research Council of Australia, and the Juvenile Diabetes Research Foundation.

## Conflict of Interest

The authors declare that the research was conducted in the absence of any commercial or financial relationships that could be construed as a potential conflict of interest.
